# Supplementary material for: Hepatitis B care cascade among people with HIV/HBV coinfection in the North American AIDS Cohort Collaboration on Research and Design, 2012–2016
Source: PLoS One. 2023 Sep 1;18(9):e0290889. doi: 10.1371/journal.pone.0290889 (PMC10473492; doi:10.1371/journal.pone.0290889)
Supplement: S1 Checklist — (DOCX) [file pone.0290889.s001.docx]

STROBE Statement—checklist of items that should be included in reports of observational studies

|  | Item No. | Recommendation | Page  No. | Relevant text from manuscript |
| --- | --- | --- | --- | --- |
| **Title and abstract** | 1 | (*a*) Indicate the study’s design with a commonly used term in the title or the abstract | Abstract, page 3 | “We conducted a cross-sectional study…” |
|  |  | (*b*) Provide in the abstract an informative and balanced summary of what was done and what was found | 3 | “We conducted a cross-sectional study among people living with HIV and HBV coinfection receiving care between January 1, 2012 and December 31, 2016 within 13 United States and Canadian clinical cohorts contributing data to the North American AIDS Cohort Collaboration on Research and Design (NA-ACCORD). We evaluated each of the steps in this cascade, including: 1) laboratory-confirmed HBV infection, 2) tenofovir-based or entecavir-based HBV therapy prescribed, 3) HBV DNA measured during treatment, and 4) viral suppression achieved via undetectable HBV DNA. Among 3,953 persons with laboratory-confirmed HBV (median age, 50 years; 6.5% female; 43.8% were Black; 7.1% were Hispanic), 3,592 (90.9%; 95% confidence interval, 90.0-91.8%) were prescribed tenofovir-based antiretroviral therapy or entecavir along with their antiretroviral therapy regimen, 2,281 (57.7%; 95% confidence interval , 56.2-59.2%) had HBV DNA measured while on therapy, and 1,624 (41.1%; 95% confidence interval, 39.5-42.6) achieved an undetectable HBV DNA during HBV treatment. Our study identified significant gaps in measurement of HBV DNA and suppression of HBV viremia among people living with HIV and HBV coinfection in the United States and Canada.” |
| Introduction | | | |  |
| Background/ rationale | 2 | Explain the scientific background and rationale for the investigation being reported | 4 | “Hepatitis B virus (HBV) infection is common among people with HIV (PWH), with a prevalence ranging from 5-15%. Research has shown that detectable HBV viremia among PWH with HBV coinfection is associated with increased rates of hepatocellular carcinoma (HCC). HBV suppression with HBV-active antiretroviral therapy (ART) reduces risk of HCC and decompensated cirrhosis. Care cascades, first used for HIV infection and later for hepatitis C virus infection, are a critical tool for evaluating delivery of care across sequential stages, starting with diagnosis and ending with viral suppression. However, there have been few data describing the HBV care cascade among PWH with HBV coinfection. This information is important to identify gaps in HBV care among PWH, target allocation of health resources to close those gaps, and promote stakeholder involvement/collaboration, which supports achievement of the 2030 World Health Organization HBV elimination goals” |
| Objectives | 3 | State specific objectives, including any prespecified hypotheses | 4 | “In this study, we describe the HBV care cascade among PWH with HBV coinfection from 2012-2016.” |
| Methods | | | |  |
| Study design | 4 | Present key elements of study design early in the paper | 4 | “We conducted a cross-sectional study among PWH with HBV coinfection receiving care between January 1, 2012 and December 31, 2016 within 13 United States (US) and Canadian clinical cohorts contributing data to the North American AIDS Cohort Collaboration on Research and Design (NA-ACCORD).” |
| Setting | 5 | Describe the setting, locations, and relevant dates, including periods of recruitment, exposure, follow-up, and data collection | 4-5 | “We conducted a cross-sectional study among PWH with HBV coinfection receiving care between January 1, 2012 and December 31, 2016 within 13 United States (US) and Canadian clinical cohorts contributing data to the North American AIDS Cohort Collaboration on Research and Design (NA-ACCORD). These cohorts include PWH who are engaged in care (≥2 HIV clinic visits within 12 months). At regular intervals, cohorts collect and securely transfer data (demographic, diagnostic, medication, sociobehavioral, laboratory, vital status information) to the Data Management Core (University of Washington) for harmonization and quality control checks. Data are transferred to the Epidemiology/Biostatistics Core (Johns Hopkins University) for additional quality checks and creation of analytic-ready files… The study was conducted between February 2022 and March 2023.”  “We focused on adult (≥18 years) PWH who were alive with HBV coinfection as of 2012 to assess HBV care when tenofovir-based ART and HBV-specific management guidelines were accessible. PWH in NA-ACCORD were eligible for inclusion if they had: 1) HBV coinfection (defined by ≥1 positive HBV surface antigen, ≥1 positive HBV e antigen, or any detectable HBV DNA) prior to December 31, 2016; 2) ≥365 days of observation in NA-ACCORD after their qualifying HBV laboratory test between January 1, 2012 and December 31, 2016; and 3) ≥1 HIV RNA and CD4+ cell measurement during the 2012-2016 period (to limit inclusion to people in HIV care).” |
| Participants | 6 | (*a*) *Cohort study*—Give the eligibility criteria, and the sources and methods of selection of participants. Describe methods of follow-up  *Case-control study*—Give the eligibility criteria, and the sources and methods of case ascertainment and control selection. Give the rationale for the choice of cases and controls  *Cross-sectional study*—Give the eligibility criteria, and the sources and methods of selection of participants | 5 | “We focused on adult (≥18 years) PWH who were alive with HBV coinfection as of 2012 to assess HBV care when tenofovir-based ART and HBV-specific management guidelines were accessible. PWH in NA-ACCORD were eligible for inclusion if they had: 1) HBV coinfection (defined by ≥1 positive HBV surface antigen, ≥1 positive HBV e antigen, or any detectable HBV DNA) prior to December 31, 2016; 2) ≥365 days of observation in NA-ACCORD after their qualifying HBV laboratory test between January 1, 2012 and December 31, 2016; and 3) ≥1 HIV RNA and CD4+ cell measurement during the 2012-2016 period (to limit inclusion to people in HIV care).” |
|  |  | (*b*) *Cohort study*—For matched studies, give matching criteria and number of exposed and unexposed  *Case-control study*—For matched studies, give matching criteria and the number of controls per case | N/A | Not applicable. |
| Variables | 7 | Clearly define all outcomes, exposures, predictors, potential confounders, and effect modifiers. Give diagnostic criteria, if applicable | 5-7 | “PWH in NA-ACCORD were eligible for inclusion if they had: 1) HBV coinfection (defined by ≥1 positive HBV surface antigen, ≥1 positive HBV e antigen, or any detectable HBV DNA) prior to December 31, 2016; 2) ≥365 days of observation in NA-ACCORD after their qualifying HBV laboratory test between January 1, 2012 and December 31, 2016; and 3) ≥1 HIV RNA and CD4+ cell measurement during the 2012-2016 period (to limit inclusion to people in HIV care).” “The main steps in this cascade include: 1) laboratory-confirmed HBV infection, 2) tenofovir-based or entecavir-based HBV therapy prescribed, 3) HBV DNA measured during treatment, and 4) viral suppression achieved via undetectable HBV DNA. For each individual with laboratory-confirmed HBV infection, we determined exposure to a first-line HBV-active antiviral drug (tenofovir disoproxil fumarate [TDF], tenofovir alafenamide [TAF], or entecavir) from 2012-2016…. ART was defined as receipt of three antiretrovirals from at least two classes. We examined all available quantitative and qualitative HBV DNA results from 2012-2016. We determined age at date of first qualifying HBV test and selected the HIV RNA and CD4+ cell count closest to that date. Sex, race/ethnicity, and HIV transmission risk factors were collected at NA-ACCORD enrollment. ” |
| Data sources/ measurement | 8* | For each variable of interest, give sources of data and details of methods of assessment (measurement). Describe comparability of assessment methods if there is more than one group | 4, 6 | “…within 13 United States (US) and Canadian clinical cohorts contributing data to the North American AIDS Cohort Collaboration on Research and Design (NA-ACCORD).”  “For each individual with laboratory-confirmed HBV infection, we determined exposure to a first-line HBV-active antiviral drug (tenofovir disoproxil fumarate [TDF], tenofovir alafenamide [TAF], or entecavir) from 2012-2016. Since 2010, HIV treatment guidelines have emphasized including tenofovir in the ART regimen of those with chronic HBV and avoidance of lamivudine or emtricitabine monotherapy, which can promote development of HBV drug-resistance mutations. If tenofovir cannot be safely used, entecavir may be administered in addition to fully suppressive ART. ART was defined as receipt of three antiretrovirals from at least two classes. We examined all available quantitative and qualitative HBV DNA results from 2012-2016. We determined age at date of first qualifying HBV test and selected the HIV RNA and CD4+ cell count closest to that date. Sex, race/ethnicity, and HIV transmission risk factors were collected at NA-ACCORD enrollment.” |
| Bias | 9 | Describe any efforts to address potential sources of bias | N/A | Not applicable. |
| Study size | 10 | Explain how the study size was arrived at | 5 | “We focused on adult (≥18 years) PWH who were alive with HBV coinfection as of 2012 to assess HBV care when tenofovir-based ART and HBV-specific management guidelines were accessible. PWH in NA-ACCORD were eligible for inclusion if they had: 1) HBV coinfection (defined by ≥1 positive HBV surface antigen, ≥1 positive HBV e antigen, or any detectable HBV DNA) prior to December 31, 2016; 2) ≥365 days of observation in NA-ACCORD after their qualifying HBV laboratory test between January 1, 2012 and December 31, 2016; and 3) ≥1 HIV RNA and CD4+ cell measurement during the 2012-2016 period (to limit inclusion to people in HIV care). All eligible patients were included.” |

| Quantitative variables | 11 | Explain how quantitative variables were handled in the analyses. If applicable, describe which groupings were chosen and why | 8-9 | Groupings listed in Table 1 |
| --- | --- | --- | --- | --- |
| Statistical methods | 12 | (*a*) Describe all statistical methods, including those used to control for confounding | 6-7 | “We evaluated a diagnosis-based cascade because we sought to determine the proportion meeting each step among a denominator of PWH with laboratory-confirmed HBV coinfection. The main steps in this cascade include: 1) laboratory-confirmed HBV infection, 2) tenofovir-based or entecavir-based HBV therapy prescribed, 3) HBV DNA measured during treatment, and 4) viral suppression achieved via undetectable HBV DNA. For each individual with laboratory-confirmed HBV infection, we determined exposure to a first-line HBV-active antiviral drug (tenofovir disoproxil fumarate [TDF], tenofovir alafenamide [TAF], or entecavir) from 2012-2016… We examined all available quantitative and qualitative HBV DNA results from 2012-2016. We determined age at date of first qualifying HBV test and selected the HIV RNA and CD4+ cell count closest to that date. Sex, race/ethnicity, and HIV transmission risk factors were collected at NA-ACCORD enrollment. We described the diagnosis-based HBV care cascade as follows:  Step 1 (HBV-Infected): Among eligible PWH in NA-ACCORD receiving HIV care between 2012-2016, we determined the number with laboratory-confirmed HBV infection.  Step 2 (Received Tenofovir or Entecavir): We determined the proportion (with 95% confidence intervals [CI]) of PWH with HBV coinfection who, between 2012-2016, received tenofovir-based ART (TDF or TAF) or entecavir along with an ART regimen.  Step 3 (HBV DNA Assessed): We determined the proportion (with 95% CI) of PWH with HBV coinfection who had any HBV DNA test (quantitative or qualitative) performed while on HBV treatment (defined in Step 2) between 2012-2016.  Step 4 (Undetectable HBV DNA): We determined the proportion (with 95% CI) of PWH with HBV coinfection who had an undetectable quantitative (HBV DNA <200 international units [IU]/mL) or qualitative (negative) HBV DNA test during HBV treatment (defined in Step 2) between 2012-2016.” |
|  |  | (*b*) Describe any methods used to examine subgroups and interactions | N/A | Not applicable. |
|  |  | (*c*) Explain how missing data were addressed |  | “We described the diagnosis-based HBV care cascade as follows:  Step 1 (HBV-Infected): Among eligible PWH in NA-ACCORD receiving HIV care between 2012-2016, we determined the number with laboratory-confirmed HBV infection.  Step 2 (Received Tenofovir or Entecavir): We determined the proportion (with 95% confidence intervals [CI]) of PWH with HBV coinfection who, between 2012-2016, received tenofovir-based ART (TDF or TAF) or entecavir along with an ART regimen.  Step 3 (HBV DNA Assessed): We determined the proportion (with 95% CI) of PWH with HBV coinfection who had any HBV DNA test (quantitative or qualitative) performed while on HBV treatment (defined in Step 2) between 2012-2016.  Step 4 (Undetectable HBV DNA): We determined the proportion (with 95% CI) of PWH with HBV coinfection who had an undetectable quantitative (HBV DNA <200 international units [IU]/mL) or qualitative (negative) HBV DNA test during HBV treatment (defined in Step 2) between 2012-2016.” |
|  |  | (*d*) *Cohort study*—If applicable, explain how loss to follow-up was addressed  *Case-control study*—If applicable, explain how matching of cases and controls was addressed  *Cross-sectional study*—If applicable, describe analytical methods taking account of sampling strategy | N/A | Not applicable. |
|  |  | (*e*) Describe any sensitivity analyses | N/A | Not applicable. |
| Results | | | | |
| Participants | 13* | (a) Report numbers of individuals at each stage of study—eg numbers potentially eligible, examined for eligibility, confirmed eligible, included in the study, completing follow-up, and analysed | 7-8, Figure 1 | “Between January 1, 2012 and December 31, 2016, there were 85,546 PWH in care within 13 clinical cohorts in NA-ACCORD. Within this sample, 73,860 (86.3%) PWH ever had HBV laboratory testing, and 5,485 (6.4%) had laboratory-confirmed HBV coinfection. After excluding those with <365 days of observation after the qualifying HBV laboratory test (n=210) or no available HIV RNA and CD4+ cell measurements during the observation period (n=1,295), 3,953 people with HIV/HBV coinfection remained in the final sample (Fig 1).” |
|  |  | (b) Give reasons for non-participation at each stage | N/A | Not applicable. |
|  |  | (c) Consider use of a flow diagram | 8, Figure 1 | Figure 1 |
| Descriptive data | 14* | (a) Give characteristics of study participants (eg demographic, clinical, social) and information on exposures and potential confounders | 7-9, Table 1 | “These individuals had a median age of 50 (interquartile range [IQR], 43-57) years, 6.5% were female, 43.8% were Black, and 7.1% were Hispanic (Table 1).” |
|  |  | (b) Indicate number of participants with missing data for each variable of interest | 8, Table 1 | Table 1 |
|  |  | (c) *Cohort study*—Summarise follow-up time (eg, average and total amount) | N/A | Not applicable. |
| Outcome data | 15* | *Cohort study*—Report numbers of outcome events or summary measures over time | N/A | Not applicable. |
|  |  | *Case-control study—*Report numbers in each exposure category, or summary measures of exposure | N/A | Not applicable. |
|  |  | *Cross-sectional study—*Report numbers of outcome events or summary measures | 10, Figure 2 | “Among the 3,953 PWH with HBV coinfection, 3,592 (90.9%; 95% CI, 90.0-91.8%) were prescribed tenofovir-based ART or entecavir along with their ART regimen (Table 1; median time on HBV therapy, 2.7 [IQR, 1.5-4.1] years), 2,281 (57.7%; 95% CI, 56.2-59.2%) had HBV DNA measured while on therapy, and 1,624 (41.1%; 95% CI, 39.5-42.6) achieved an undetectable HBV DNA during HBV treatment (Fig 2). Among those who had HBV DNA measured, 1,624 (71.2%) achieved HBV suppression on therapy.” |
| Main results | 16 | (*a*) Give unadjusted estimates and, if applicable, confounder-adjusted estimates and their precision (eg, 95% confidence interval). Make clear which confounders were adjusted for and why they were included | 10, Figure 2 | “Among the 3,953 PWH with HBV coinfection, 3,592 (90.9%; 95% CI, 90.0-91.8%) were prescribed tenofovir-based ART or entecavir along with their ART regimen (Table 1; median time on HBV therapy, 2.7 [IQR, 1.5-4.1] years), 2,281 (57.7%; 95% CI, 56.2-59.2%) had HBV DNA measured while on therapy, and 1,624 (41.1%; 95% CI, 39.5-42.6) achieved an undetectable HBV DNA during HBV treatment (Fig 2). Among those who had HBV DNA measured, 1,624 (71.2%) achieved HBV suppression on therapy.” |
|  |  | (*b*) Report category boundaries when continuous variables were categorized | 8, Table 1 | Table 1 |
|  |  | (*c*) If relevant, consider translating estimates of relative risk into absolute risk for a meaningful time period | N/A | Not applicable. |

| Other analyses | 17 | Report other analyses done—eg analyses of subgroups and interactions, and sensitivity analyses | N/A | Not applicable. |
| --- | --- | --- | --- | --- |
| Discussion | | | | |
| Key results | 18 | Summarise key results with reference to study objectives | 10-12 | “In this study, we identified gaps in HBV-related management among PWH with HBV coinfection in care in the US and Canada. The largest drop-offs occurred in assessment of HBV DNA and confirmation of HBV suppression on HBV-active therapy. Our study highlights opportunities for improving HBV-related care among PWH with HBV coinfection in a population known to be at risk for more accelerated liver disease progression. We observed that 9.0% of PWH with HBV coinfection did not receive either tenofovir-based ART or entecavir along with their ART regimen. The absence of anti-HBV therapy represents a missed opportunity for prevention of liver complications. We found that only 57.7% of PWH with HBV coinfection were tested for HBV DNA while on HBV therapy during the observation period… Despite the increased risk of liver complications with elevated HBV DNA levels, only 41.1% of PWH with HBV coinfection on HBV-active ART were confirmed to achieve an undetectable HBV DNA. Among PWH with HBV coinfection who had HBV DNA measured, 71.2% had confirmed HBV suppression... Therefore, to ensure the maximal protective benefits from HBV-active ART, providers should be aware of the importance of assessing HBV DNA and confirming HBV suppression. Integrating reminders to measure HBV DNA in electronic medical record systems, creating automated order sets, and provider education may help increase HBV DNA assessments in clinical care.” |
| Limitations | 19 | Discuss limitations of the study, taking into account sources of potential bias or imprecision. Discuss both direction and magnitude of any potential bias | 12 | “Our study has several potential limitations. Adefovir or telbivudine may be prescribed for HBV treatment, but these antivirals are not collected by NA-ACCORD. However, they are used infrequently in most settings, particularly among PWH. Second, this study utilized data from 2012-2016 and may not entirely represent current practice. However, HBV management guidelines in HIV have remained largely unchanged during the intervening time, arguing for continued relevance of trends observed in this cascade of care. Finally, NA-ACCORD cohorts included in this analysis represent US and Canadian demographics of PWH in care.” |
| Interpretation | 20 | Give a cautious overall interpretation of results considering objectives, limitations, multiplicity of analyses, results from similar studies, and other relevant evidence | 10-12 | “…Our study identified significant gaps in measurement of HBV DNA and suppression of HBV viremia among PWH with HBV coinfection in the US and Canada. Periodic evaluation of the HBV care cascade among persons with HIV/HBV will be critical to monitoring success in completion of each step.” |
| Generalisability | 21 | Discuss the generalisability (external validity) of the study results | 12 | “NA-ACCORD cohorts included in this analysis represent US and Canadian demographics of PWH in care.” |
| Other information | |  | | |
| Funding | 22 | Give the source of funding and the role of the funders for the present study and, if applicable, for the original study on which the present article is based | Editorial Manager | Per PLOS ONE’s guidelines, this information is not in the manuscript text, but is included in the online Editorial Manager submission: “This work was supported by a research grant from the National Institute of Allergy and Infectious Diseases [grant number R21 AI124868 (VLR)]. Additional support was provided by National Institutes of Health [grant numbers U01 AI069918 (KNA, RDM), F31 AI124794, F31 DA037788, G12 MD007583, K01 AI093197 (KNA), K01 AI131895, K08 DK132977 (JT), K23 EY013707, K24 AI065298 (TRS), K24 AI118591, K24 DA000432 (RDM), KL2 TR000421, N01 CP01004, N02 CP055504, N02 CP91027, P30 AI027757, P30 AI027763, P30 AI027767, P30 AI036219, P30 AI050409, P30 AI050410, P30 AI094189, P30 AI110527, P30 MH62246, R01 AA016893 (RDM), R01 DA011602 (RDM), R01 DA012568, R01 AG053100 (KNA), R24 AI067039, U01 AA013566, U01 AA020790, U01 AA020793, U01 AI038855, U01 AI038858, U01 AI068634, U01 AI068636, U01 AI069432, U01 AI069434, U01 DA036297 (GDK), R01 DA048063, U01 DA03629, U01 DA036935 (RDM), U10 EY008057, U10 EY008052, U10 EY008067, U01 HL146192, U01 HL146193, U01 HL146194, U01 HL146201, U01 HL146202, U01 HL146203, U01 HL146204, U01 HL146205, U01 HL146208, U01 HL146240, U01 HL146241, U01 HL146242, U01 HL146245, U01 HL146333, U24 AA020794, U54 GM133807, UL1 RR024131, UL1 TR000004, UL1 TR000083, Z01 CP010214 and Z01 CP010176]; Centers for Disease Control and Prevention, USA [contract numbers CDC-200-2006-18797 and CDC-200-2015-63931]; Agency for Healthcare Research and Quality, USA [contract number 90047713]; Health Resources and Services Administration, USA [contract number 90051652]; Canadian Institutes of Health Research, Canada [grant numbers CBR 86906, CBR 94036, HCP 97105 and TGF 96118]; Ontario Ministry of Health and Long Term Care; and the Government of Alberta, Canada. Additional support was provided by the National Institute of Allergy and Infectious Diseases (NIAID), National Cancer Institute (NCI), National Heart, Lung, and Blood Institute (NHLBI), Eunice Kennedy Shriver National Institute of Child Health & Human Development (NICHD), National Human Genome Research Institute (NHGRI), National Institute for Mental Health (NIMH) and National Institute on Drug Abuse (NIDA), National Institute on Aging (NIA), National Institute of Dental & Craniofacial Research (NIDCR), National Institute of Neurological Disorders And Stroke (NINDS), National Institute of Nursing Research (NINR), National Institute on Alcohol Abuse and Alcoholism (NIAAA), National Institute on Deafness and Other Communication Disorders (NIDCD), and National Institute of Diabetes and Digestive and Kidney Diseases (NIDDK). These data were collected by cancer registries participating in the National Program of Cancer Registries (NPCR) of the Centers for Disease Control and Prevention (CDC). The funders had no role in study design, data collection and analysis, decision to publish, or preparation of the manuscript.” |

*Give information separately for cases and controls in case-control studies and, if applicable, for exposed and unexposed groups in cohort and cross-sectional studies.

**Note:** An Explanation and Elaboration article discusses each checklist item and gives methodological background and published examples of transparent reporting. The STROBE checklist is best used in conjunction with this article (freely available on the Web sites of PLoS Medicine at http://www.plosmedicine.org/, Annals of Internal Medicine at http://www.annals.org/, and Epidemiology at http://www.epidem.com/). Information on the STROBE Initiative is available at www.strobe-statement.org.
